# Supplementary material for: Electronic Properties of Cyclacenes from TAO-DFT
Source: Sci Rep. 2016 Nov 17;6:37249. doi: 10.1038/srep37249 (PMC5112520; doi:10.1038/srep37249)
Supplement: Supplementary Information [file srep37249-s1.pdf]

Supplementary Material to: Electronic Properties of Cyclacenes from TAO-DFT

Chun-Shian Wu,[1,2,†] Pei-Yin Lee,[1,†] and Jeng-Da Chai[1,3,*]

[1] *Department of Physics, National Taiwan University, Taipei 10617, Taiwan*

[2] *Department of Chemistry, National Taiwan University, Taipei 10617, Taiwan*

[3] *Center for Theoretical Sciences and Center for Quantum Science and Engineering, National Taiwan University, Taipei 10617, Taiwan*

[†] These authors contributed equally to this work.

[*] Author to whom correspondence should be addressed. Electronic mail: jdchai@phys.ntu.edu.tw

LIST OF TABLES

S1 Singlet-triplet energy gap (in kcal/mol) of n-cyclacene as a function of the number of benzene rings, calculated using TAO-LDA and KS-LDA. For comparison, the CASPT2, KS-M06L, and KS-B3LYP data are taken from the literature [1]. 3

S2 Singlet-triplet energy gap (in kcal/mol) of n-cyclacene/n-acene as a function of the number of benzene rings, calculated using TAO-LDA. 3

S3 Vertical ionization potential (in eV) for the lowest singlet state of n-cyclacene/n-acene as a function of the number of benzene rings, calculated using TAO-LDA. 7

S4 Vertical electron affinity (in eV) for the lowest singlet state of n-cyclacene/n-acene as a function of the number of benzene rings, calculated using TAO-LDA. 10

S5 Fundamental gap (in eV) for the lowest singlet state of n-cyclacene/n-acene as a function of the number of benzene rings, calculated using TAO-LDA. . . . 14

S6 Symmetrized von Neumann entropy for the lowest singlet state of n-cyclacene/n-acene as a function of the number of benzene rings, calculated using TAO-LDA. 17

[1] Sadowsky, D., McNeill, K. & Cramer, C. J. Electronic structures of [n]-cyclacenes (n = 6-12) and short, hydrogen-capped, carbon nanotubes. *Farad. Discuss*, **145**, 507–521 (2010).

TABLES

TABLE S1. Singlet-triplet energy gap (in kcal/mol) of n-cyclacene as a function of the number of benzene rings, calculated using TAO-LDA and KS-LDA. For comparison, the CASPT2, KS-M06L, and KS-B3LYP data are taken from the literature [1].

n	TAO-LDA	KS-LDA	CASPT2	KS-M06L	KS-B3LYP
4	36.72	40.93			
5	6.01	7.52			
6	9.96	8.95	12.00	12.31	15.97
7	6.10	10.45	12.00	7.70	9.87
8	11.14	10.02	14.51	13.10	21.85
9	2.84	3.32	8.01	4.59	6.93
10	9.02	10.78	11.40	13.60	54.76
11	2.49	2.64	7.50	3.80	6.67
12	6.35	11.31	13.77	10.99	
13	2.77	2.74			
14	4.28	10.81			
15	3.04	3.43			
16	3.09	8.13			
17	3.06	4.69			
18	2.52	4.51			
19	2.83	6.16			
20	2.25	2.86			

TABLE S2. Singlet-triplet energy gap (in kcal/mol) of n-cyclacene/n-acene as a function of the number of benzene rings, calculated using TAO-LDA.

n	n-cyclacene	n-acene
4	36.72	29.01
5	6.01	19.60
6	9.96	13.55
7	6.10	9.91
8	11.14	7.85
9	2.84	6.66
10	9.02	5.91
11	2.49	5.32
12	6.35	4.82
13	2.77	4.38
14	4.28	3.98
15	3.04	3.65
16	3.09	3.37
17	3.06	3.14
18	2.52	2.94
19	2.83	2.77
20	2.25	2.62
21	2.51	2.49
22	2.11	2.37
23	2.21	2.25
24	2.00	2.15
25	1.97	2.06
26	1.89	1.98
27	1.80	1.90
28	1.77	1.83
29	1.67	1.76
30	1.65	1.70

31	1.57	1.64
32	1.54	1.59
33	1.48	1.54
34	1.44	1.49
35	1.40	1.45
36	1.36	1.40
37	1.32	1.36
38	1.28	1.33
39	1.25	1.29
40	1.22	1.26
41	1.19	1.23
42	1.16	1.20
43	1.14	1.17
44	1.11	1.14
45	1.09	1.11
46	1.06	1.09
47	1.04	1.07
48	1.02	1.04
49	1.00	1.02
50	0.98	1.00
51	0.96	0.98
52	0.93	0.96
53	0.92	0.94
54	0.90	0.92
55	0.89	0.91
56	0.87	0.89
57	0.86	0.87
58	0.84	0.86
59	0.83	0.84
60	0.81	0.83

61	0.80	0.82
62	0.79	0.80
63	0.77	0.79
64	0.76	0.78
65	0.75	0.76
66	0.74	0.75
67	0.73	0.74
68	0.72	0.73
69	0.71	0.72
70	0.70	0.71
71	0.69	0.70
72	0.68	0.69
73	0.67	0.68
74	0.66	0.67
75	0.65	0.66
76	0.64	0.65
77	0.63	0.64
78	0.63	0.64
79	0.62	0.63
80	0.61	0.62
81	0.60	0.61
82	0.60	0.60
83	0.59	0.60
84	0.58	0.59
85	0.57	0.58
86	0.57	0.58
87	0.56	0.57
88	0.55	0.56
89	0.55	0.56
90	0.54	0.55

91	0.54	0.54
92	0.53	0.54
93	0.52	0.53
94	0.52	0.53
95	0.51	0.52
96	0.51	0.51
97	0.50	0.51
98	0.50	0.50
99	0.49	0.50
100	0.49	0.49

TABLE S3. Vertical ionization potential (in eV) for the lowest singlet state of n-cyclacene/n-acene as a function of the number of benzene rings, calculated using TAO-LDA.

n	n-cyclacene	n-acene
4	6.99	6.46
5	5.95	6.07
6	5.73	5.79
7	5.53	5.59
8	5.59	5.44
9	5.33	5.33
10	5.41	5.23
11	5.21	5.15
12	5.24	5.08
13	5.12	5.01
14	5.10	4.96
15	5.04	4.91
16	4.99	4.86
17	4.96	4.82
18	4.91	4.78

19	4.89	4.75
20	4.84	4.71
21	4.82	4.68
22	4.78	4.66
23	4.76	4.63
24	4.73	4.61
25	4.71	4.59
26	4.69	4.57
27	4.67	4.55
28	4.65	4.53
29	4.63	4.51
30	4.61	4.50
31	4.59	4.48
32	4.57	4.47
33	4.56	4.45
34	4.54	4.44
35	4.53	4.43
36	4.52	4.42
37	4.50	4.40
38	4.49	4.39
39	4.48	4.38
40	4.47	4.37
41	4.45	4.36
42	4.44	4.35
43	4.43	4.35
44	4.42	4.34
45	4.41	4.33
46	4.40	4.32
47	4.40	4.31
48	4.39	4.31

49	4.38	4.30
50	4.37	4.29
51	4.36	4.29
52	4.36	4.28
53	4.35	4.27
54	4.34	4.27
55	4.33	4.26
56	4.33	4.26
57	4.32	4.25
58	4.32	4.24
59	4.31	4.24
60	4.30	4.23
61	4.30	4.23
62	4.29	4.23
63	4.29	4.22
64	4.28	4.22
65	4.28	4.21
66	4.27	4.21
67	4.27	4.20
68	4.26	4.20
69	4.26	4.20
70	4.25	4.19
71	4.25	4.19
72	4.24	4.18
73	4.24	4.18
74	4.23	4.18
75	4.23	4.17
76	4.23	4.17
77	4.22	4.17
78	4.22	4.16

79	4.22	4.16
80	4.21	4.16
81	4.21	4.15
82	4.20	4.15
83	4.20	4.15
84	4.20	4.15
85	4.19	4.14
86	4.19	4.14
87	4.19	4.14
88	4.18	4.14
89	4.18	4.13
90	4.18	4.13
91	4.18	4.13
92	4.17	4.13
93	4.17	4.12
94	4.17	4.12
95	4.16	4.12
96	4.16	4.12
97	4.16	4.11
98	4.16	4.11
99	4.15	4.11
100	4.15	4.11

TABLE S4. Vertical electron affinity (in eV) for the lowest singlet state of n-cyclacene/n-acene as a function of the number of benzene rings, calculated using TAO-LDA.

n	n-cyclacene	n-acene
4	0.19	0.90
5	1.23	1.34
6	1.17	1.66
7	1.45	1.89

8	1.53	2.06
9	1.88	2.19
10	1.88	2.30
11	2.14	2.39
12	2.15	2.48
13	2.33	2.55
14	2.36	2.61
15	2.47	2.67
16	2.52	2.72
17	2.58	2.77
18	2.64	2.81
19	2.68	2.85
20	2.73	2.89
21	2.77	2.92
22	2.81	2.95
23	2.84	2.98
24	2.88	3.00
25	2.90	3.03
26	2.93	3.05
27	2.96	3.07
28	2.98	3.09
29	3.01	3.11
30	3.03	3.13
31	3.05	3.15
32	3.07	3.16
33	3.09	3.18
34	3.11	3.19
35	3.12	3.21
36	3.14	3.22
37	3.15	3.23

38	3.17	3.25
39	3.18	3.26
40	3.19	3.27
41	3.21	3.28
42	3.22	3.29
43	3.23	3.30
44	3.24	3.31
45	3.25	3.32
46	3.26	3.33
47	3.27	3.33
48	3.28	3.34
49	3.29	3.35
50	3.30	3.36
51	3.31	3.36
52	3.32	3.37
53	3.32	3.38
54	3.33	3.39
55	3.34	3.39
56	3.35	3.40
57	3.35	3.40
58	3.36	3.41
59	3.37	3.42
60	3.37	3.42
61	3.38	3.43
62	3.39	3.43
63	3.39	3.44
64	3.40	3.44
65	3.40	3.45
66	3.41	3.45
67	3.41	3.46

68	3.42	3.46
69	3.42	3.46
70	3.43	3.47
71	3.43	3.47
72	3.44	3.48
73	3.44	3.48
74	3.45	3.48
75	3.45	3.49
76	3.46	3.49
77	3.46	3.50
78	3.46	3.50
79	3.47	3.50
80	3.47	3.51
81	3.48	3.51
82	3.48	3.51
83	3.48	3.52
84	3.49	3.52
85	3.49	3.52
86	3.49	3.52
87	3.50	3.53
88	3.50	3.53
89	3.50	3.53
90	3.51	3.54
91	3.51	3.54
92	3.51	3.54
93	3.52	3.54
94	3.52	3.55
95	3.52	3.55
96	3.52	3.55
97	3.53	3.55

98	3.53	3.56
99	3.53	3.56
100	3.53	3.56

TABLE S5. Fundamental gap (in eV) for the lowest singlet state of n-cyclacene/n-acene as a function of the number of benzene rings, calculated using TAO-LDA.

n	n-cyclacene	n-acene
4	6.81	5.56
5	4.72	4.73
6	4.56	4.13
7	4.08	3.69
8	4.06	3.38
9	3.45	3.13
10	3.53	2.93
11	3.07	2.76
12	3.09	2.60
13	2.79	2.46
14	2.74	2.34
15	2.57	2.23
16	2.47	2.13
17	2.38	2.05
18	2.27	1.97
19	2.21	1.89
20	2.11	1.83
21	2.05	1.76
22	1.97	1.71
23	1.92	1.65
24	1.86	1.60
25	1.81	1.56
26	1.75	1.52

27	1.71	1.47
28	1.66	1.44
29	1.62	1.40
30	1.58	1.37
31	1.54	1.33
32	1.50	1.30
33	1.47	1.27
34	1.44	1.25
35	1.41	1.22
36	1.38	1.20
37	1.35	1.17
38	1.32	1.15
39	1.30	1.13
40	1.27	1.11
41	1.25	1.09
42	1.22	1.07
43	1.20	1.05
44	1.18	1.03
45	1.16	1.01
46	1.14	1.00
47	1.12	0.98
48	1.11	0.96
49	1.09	0.95
50	1.07	0.94
51	1.05	0.92
52	1.04	0.91
53	1.02	0.89
54	1.01	0.88
55	0.99	0.87
56	0.98	0.86

57	0.97	0.85
58	0.95	0.84
59	0.94	0.82
60	0.93	0.81
61	0.92	0.80
62	0.91	0.79
63	0.89	0.78
64	0.88	0.77
65	0.87	0.77
66	0.86	0.76
67	0.85	0.75
68	0.84	0.74
69	0.83	0.73
70	0.82	0.72
71	0.81	0.72
72	0.80	0.71
73	0.80	0.70
74	0.79	0.69
75	0.78	0.69
76	0.77	0.68
77	0.76	0.67
78	0.75	0.67
79	0.75	0.66
80	0.74	0.65
81	0.73	0.65
82	0.72	0.64
83	0.72	0.63
84	0.71	0.63
85	0.70	0.62
86	0.70	0.62

87	0.69	0.61
88	0.69	0.61
89	0.68	0.60
90	0.67	0.59
91	0.67	0.59
92	0.66	0.58
93	0.66	0.58
94	0.65	0.58
95	0.64	0.57
96	0.64	0.57
97	0.63	0.56
98	0.63	0.56
99	0.62	0.55
100	0.62	0.55

TABLE S6. Symmetrized von Neumann entropy for the lowest singlet state of n-cyclacene/n-acene as a function of the number of benzene rings, calculated using TAO-LDA.

n	n-cyclacene	n-acene
4	0.04	0.15
5	1.51	0.40
6	1.03	0.75
7	1.53	1.08
8	0.99	1.34
9	2.50	1.52
10	1.23	1.67
11	2.80	1.82
12	1.74	1.98
13	2.83	2.16
14	2.42	2.36
15	2.90	2.55

16	3.06	2.75
17	3.10	2.94
18	3.55	3.13
19	3.43	3.31
20	3.92	3.50
21	3.85	3.69
22	4.24	3.88
23	4.29	4.07
24	4.55	4.26
25	4.72	4.44
26	4.90	4.63
27	5.11	4.82
28	5.26	5.01
29	5.49	5.20
30	5.64	5.39
31	5.86	5.58
32	6.03	5.77
33	6.23	5.95
34	6.41	6.14
35	6.60	6.33
36	6.79	6.52
37	6.98	6.71
38	7.17	6.90
39	7.36	7.09
40	7.55	7.27
41	7.73	7.46
42	7.92	7.65
43	8.11	7.84
44	8.30	8.03
45	8.49	8.22

46	8.68	8.41
47	8.87	8.59
48	9.06	8.78
49	9.24	8.97
50	9.43	9.16
51	9.62	9.35
52	9.81	9.54
53	10.00	9.73
54	10.19	9.92
55	10.38	10.10
56	10.56	10.29
57	10.75	10.48
58	10.94	10.67
59	11.13	10.86
60	11.32	11.05
61	11.51	11.23
62	11.70	11.42
63	11.89	11.61
64	12.07	11.80
65	12.26	11.99
66	12.45	12.18
67	12.64	12.37
68	12.83	12.56
69	13.02	12.74
70	13.21	12.93
71	13.39	13.12
72	13.58	13.31
73	13.77	13.50
74	13.96	13.69
75	14.15	13.88

76	14.34	14.06
77	14.53	14.25
78	14.71	14.44
79	14.90	14.63
80	15.09	14.82
81	15.28	15.01
82	15.47	15.20
83	15.66	15.38
84	15.85	15.57
85	16.04	15.76
86	16.22	15.95
87	16.41	16.14
88	16.60	16.33
89	16.79	16.52
90	16.98	16.70
91	17.17	16.89
92	17.36	17.08
93	17.54	17.27
94	17.73	17.46
95	17.92	17.65
96	18.11	17.84
97	18.30	18.02
98	18.49	18.21
99	18.68	18.40
100	18.86	18.59
